# Examining the impact of cross-domain learning on crime prediction

**DOI:** 10.1186/s40537-021-00489-9

**Published:** 2021-07-03

**Authors:** Fateha Khanam Bappee, Amilcar Soares, Lucas May Petry, Stan Matwin

**Affiliations:** 1 Faculty of Computer Science, Dalhousie University, Halifax, Nova Scotia Canada; 2 Department of Computer Science, Memorial University of Newfoundland, St. John’s, Canada; 3 Universidade Federal de Santa Catarina, Florianópolis, Brazil; 4 Institute of Computer Science, Polish Academy of Sciences, Warsaw, Poland

**Keywords:** Crime prediction, Data-driven approach, Domain adaptation, Transfer learning

## Abstract

Nowadays, urban data such as demographics, infrastructure, and criminal records are becoming more accessible to researchers. This has led to improvements in quantitative crime research for predicting future crime occurrence by identifying factors and knowledge from instances that contribute to criminal activities. While crime distribution in the geographic space is asymmetric, there are often analog, implicit criminogenic factors hidden in the data. And, since the data are not as available or comprehensive, especially for smaller cities, it is challenging to build a uniform framework for all geographic regions. This paper addresses the crime prediction task from a cross-domain perspective to tackle the data insufficiency problem in a small city. We create a uniform outline for Halifax, Nova Scotia, one of Canada’s geographic regions, by adapting and learning knowledge from two different domains, Toronto and Vancouver, which belong to different but related distributions with Halifax. For transferring knowledge among source and target domains, we propose applying instance-based transfer learning settings. Each setting is directed to learning knowledge based on a seasonal perspective with cross-domain data fusion. We choose ensemble learning methods for model building as it has generalization capabilities over new data. We evaluate the classification performance for both single and multi-domain representations and compare the results with baseline models. Our findings exhibit the satisfactory performance of our proposed data-driven approach by integrating multiple sources of data.

## Introduction

The economic development of a country and the quality of its civic life are subject to urban safety and security. These days, researchers are highly motivated to address the challenges of urban crime research and crime prediction problems due to the availability of cutting-edge technologies in big data analytics and machine learning. In general, urban profiling links to comprehensive, dynamic, and diverse patterns for each neighborhood. After, these patterns must be efficiently solved computationally to gain the highest benefit.

With the increasing accessibility of crowd-sourced and open data in big cities, there has been an interest in applying domain adaptation and transfer learning techniques across cities and transferring knowledge from a big to a small city [1]. Recently, the thought of knowledge transfer among different domains has been applied effectively in numerous real-world applications [2, 3]. Several studies have explored transfer learning in a renowned machine learning field known as natural language processing (NLP) [4–6]. Liu et al. [1] introduced a domain adaptation network to identify parking hotspots with shared bikes for Beijing by utilizing Shanghai’s knowledge. Another study investigated transfer learning for building predictive models of C. difficile infection by using information from multiple hospitals [7]. Nevertheless, only limited research [8, 9] has been done to explore transfer learning and domain adaptation in crime prediction.

With significant advances in machine learning techniques, it is possible to promote crime research with prominent urban features. Existing data-driven crime research mostly addresses big cities with dense and diverse characteristics [10–12]. However, demography, urbanization, and societal factors differ by region and city size. Due to data insufficiency, privacy concerns, and geographically asymmetric crime data distribution, it is challenging to develop a uniform outline for all regions in a small city. Besides, population movements and commuting facilities between cities and cross-city interoperability features motivate us to leverage transfer learning techniques with the crime prediction problem.

In our definition, a small city refers to a city with low or medium population density, i.e., under 1 million [13]. On this premise, we based our research on a small city such as Halifax (population: 403,131 in 2016) [14], Nova Scotia, Canada, to gain an inherent understanding of its physical and human impact characteristics.

**Fig. 1:**
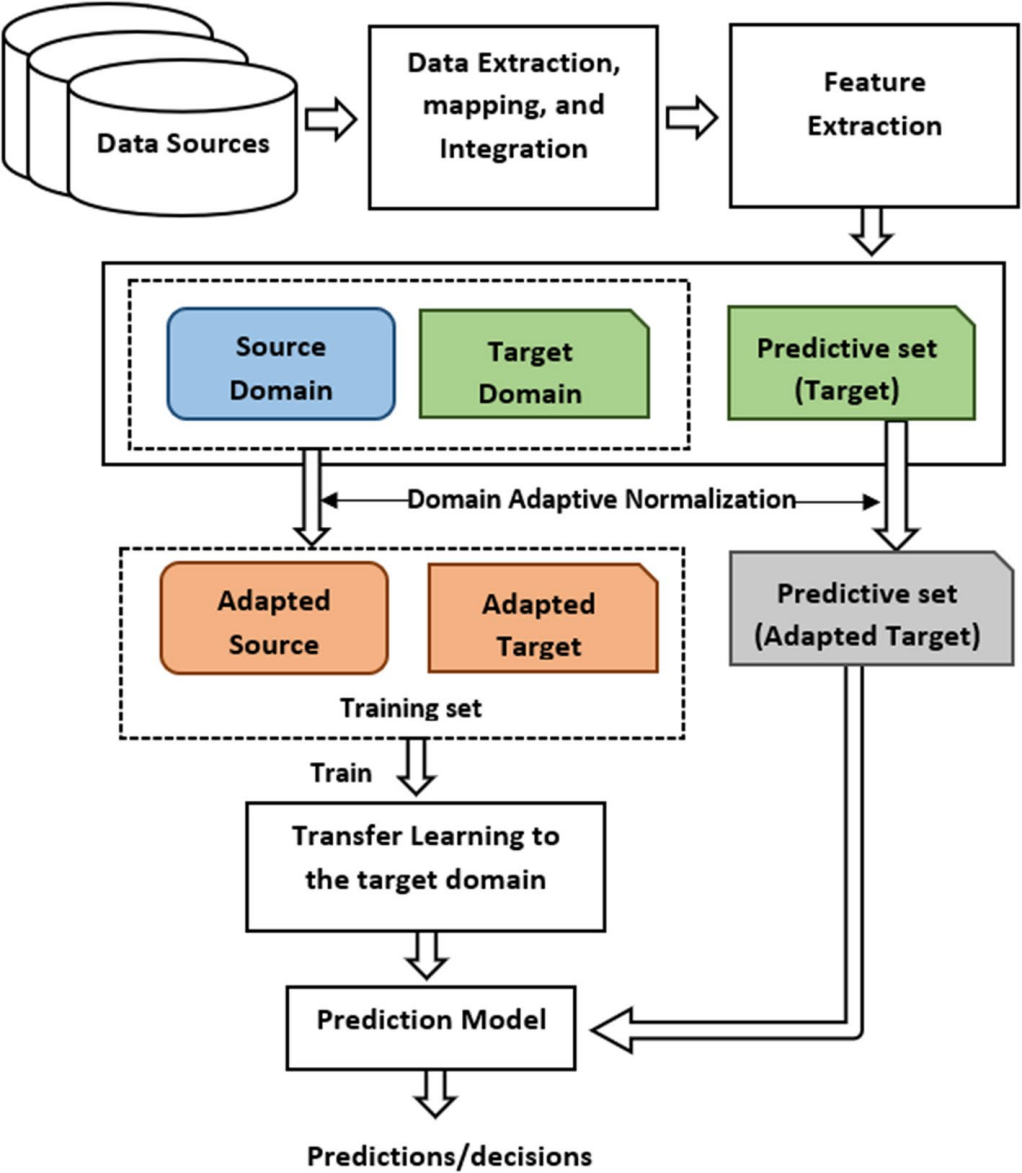
The framework of our proposed cross-domain learning approach for crime prediction task. The source and target domains are defined by the rectangle rounded corner and single corner snipped shapes, respectively

We formulate our research from a transfer learning and domain adaptation point of view, considering the challenges of preparing a satisfactory amount of labeled training data for crime prediction built on our target city (i.e., Halifax). We propose multi-source domain adaptation techniques by adapting different domains (e.g., the cities of Toronto and Vancouver) data to be used in Halifax (i.e., different but related distributions). For domain adaptation, we apply local and global min-max normalization techniques. For transferring knowledge among different domains, we consider instance-transfer to learn informative instances by seasonal subset selection. We represent different transfer learning scenarios based on the seasonal perspective with cross-domain data fusion. We tested all setups with Gradient Boosting (GB) classifiers and compared the results with the Random Forest (RF) method and some well-known ensemble-based transfer learning methods. The results show GB’s satisfactory performance for crime prediction by incorporating Toronto and Vancouver domains’ data to Halifax. The general framework for addressing the crime prediction task from a cross-domain perspective is presented in Fig. 1.

This paper’s contributions are: (i) we propose to apply supervised domain adaptation and transfer learning approaches to urban crime data. To the best of our knowledge, this work is the first to join crime research with transfer learning research. We analyze multiple sources of domains to find out the related source domain with the target and to increase positive knowledge transfer for the target domain; (ii) we study instance-based transfer learning methods and propose a seasonality-based subset selection method for transferring knowledge of instances; and (iii) we show that ensemble machine learning techniques can use generalization for different but related domains. We evaluate how an ensemble learning method such as Gradient Boosting (GB) outperforms the baselines on crime prediction in three cities.

To evaluate our proposed transfer learning strategies combined with GB, we compare our results with two widely used boosting-based transfer learning methods named TrAdaBoost [15] and TrResampling [16]. No existing research addressed the crime prediction problem from transfer learning perspectives to the best of our knowledge. Therefore, we could not compare our transfer learning method with other baselines in the crime research field. We analyze the results obtained from the AUC (Area Under the ROC Curve) and Gmean scores individually for each model. The experiments performed in this work show that our proposed method outperforms the baselines.

The remainder of the paper is organized as follows. The “Related work” section provides a review of the existing works on urban crime research and factors contributing to different criminal activities and their consequences. It also summarizes some current research based on transfer learning approaches. The “Datasets” section introduces the data sources, its retrieval, and usage in our research. The “Feature extraction” section investigates an extensive set of features from different aspects. In the “Transfer learning on domain adaptation” section, the scope of domain adaptation and transfer learning on crime research are addressed. It focuses on instance-based transfer learning for predicting future crime incidents. The “Experimental setup and result analysis” section evaluates the experimental results obtained from the ensemble learning technique and compares the results with some baseline transfer learning methods.“Conclusions and future work” summarizes the overall findings, concluding remarks of the study; it also presents some future research ideas for urban crime study.

## Related work

In this paper, we divide the related work section into two different subsections. The first subsection describes the crime prediction problem from various aspects. The second subsection provides a review of the existing research on transfer learning approaches.

### Crime prediction

Existing scientific and criminology studies work on various factors such as spatiotemporal, historical, demographic, and human behavioral factors linked to different criminal activities and crime prediction tasks. We detail the works in such elements in the following subsections.

#### Temporal and historical aspect

Understanding temporal and historical aspects of different crime and criminal activities are of great importance for predictive policing. Temporal trends have been analyzed in several studies [17–20] for crime research. Bromley et al. [17] explored temporal characteristics of alcohol-related crime for Worcester, revealing a strong association of alcohol-related crime with night-time leisure zone and night-time revelers. Similarly, Cusimano et al. [21] found the relationship between ambulance dispatch and bar closing time to be from 12 am to 4 am in their study. Recently, temporal properties such as a month, year, weekday, and seasonal patterns of crime occurrence are included in [10, 22, 23], though the authors mainly focus on demographic features and mobile network activity. Several works also explore historical information and temporal knowledge to predict future crime incidents [23–25]. A proactive decision-making environment is proposed in [26] by employing the seasonal trend decomposition technique with historical knowledge.

#### Spatial aspect

As stated by the crime pattern theory of criminology, space is one of the influential factors which might significantly affect criminal occurrences [10]. Crimes are not randomly distributed throughout the space, and the geographic area of a crime may vary from one place to another. Spatial pattern analysis aims to discover the spatial distribution and aggregation of crime. Several studies analyzed spatial patterns in conjunction with some other patterns while predicting crime occurrence [11, 27–29]. For example, Yu et al. [30] proposed a global spatio-temporal pattern for crime forecasting using the Cluster-Confidence-Rate-Boosting (CCRBoost) algorithm. The algorithm iteratively picks some local patterns with a minimum classification error. Later, in [31], the authors introduced Points-Of-Interest (POI) data to extract fine-grained knowledge about a geographic region and observed a positive correlation between geographical influence features and crime rate. In another study [32], a density-based clustering algorithm, HDBSCAN (Hierarchical Density-Based Spatial Clustering of Applications with Noise), is proposed to extract hotspots. Then, the shortest distance between each hotpoint and crime point is calculated to obtain the spatial feature. Recently, a study [33] investigated spatial signatures by utilizing home security technologies and observed a significant change in Vancouver’s spatial distribution of residential burglary crime.

#### Demographic aspect

Demographic and socioeconomic features have widely been used by researchers for crime prediction [10, 22]. Previous studies have applied various demographic factors, such as population, number of vacant houses, owner-occupied houses, number of people who are married or separated [34], population density, poverty, residential stability [31, 35, 36], type of premises [37], education, ages, income levels [10, 11, 27], property values [29], etc. All these works identified a significant correlation between these factors and criminal activities. Recently, Fatehkia et al. [38] proposed leveraging Facebook ‘interests’ data from the Facebook Advertising API with demographic data for crime rate prediction. Interests are analyzed based on four different groups such as movie, game, music, and relationship-related interests for specific age groups and gender. The study found that integrating Facebook interests data with demographic census data improves the models’ prediction power.

#### Human behavioral aspect

Recently, there has been increasing interest in exploring human mobility and behavioral patterns in crime research. For example, Bogomolov et al. [22] proposed a data-driven approach to address the crime prediction problem by integrating human behavioral data with human demographics. They computed human behavioral data by utilizing mobile network activity, which is referred as the Smartsteps data. In particular, this study estimated the number of people visited in a specified smallest geographic unit or cell by summing up the total number of unique phone calls in each hour. Later, in another study, the authors investigated the human dynamics feature evolved from the same mobile network infrastructure for crime hotspots classification [27]. Wang et al. [31] introduced taxi flow data to understand city dynamics by connecting neighborhoods and non-adjacent locations. The study hypothesized that two non-adjacent communities might have a strong correlation, and the social interaction between those communities might propagate the crime rate. In [39], the authors extended this work [31] by combining both the taxi flow graph and spatial graph with learning region representations. In particular, the authors proposed a graph embedding method to uncover the relationship between urban dynamics and crime rate prediction. Similarly, several studies [10, 25, 40] incorporated dynamic city knowledge from transportation and human mobility data for crime prediction. Their data are mainly obtained from Open Street Map (OSM), Transport department, and location-based social network, i.e., Foursquare. A more recent data-driven approach has been proposed in [23] for crime occurrence prediction. Instead of focusing on mega-cities, the study mainly worked on smaller cities and analyzed human behavioral data’s impact with human demographics.

The majority of the current crime research is directed to large urban communities that exhibit dense and diverse characteristics. At the same time, urban planning paradigms and societal variables contrast by locale and size of the community. The existing research did not actualize their ideas from a cross-domain learning perspective. Aiming to learn a uniform model for all cities given different data distributions, we present a cross-domain transfer learning approach for crime occurrence prediction.

### Transfer learning

In this section, three different transfer learning approaches (e.g., instance-transfer, feature-representation-transfer, and model-transfer) based on what type of knowledge is transferred across domains [2] are discussed. In general, the instance-based transfer learning setting uses instance re-weighting and resampling techniques to obtain the relevant source instances, which can then be used with the labeled target data. In recent years, many extended boosting-based ensemble learning methods have been proposed for this setting. TrAdaBoost is a widely used boosting-based transfer learning algorithm that addresses the instance transfer learning problem [15]. This method’s main goal is to train a classifier using both the old (source) and new (target) domain data and transfer knowledge between different distribution instance spaces. In this approach, old data, which is significantly dissimilar from new data and incorrectly classified, get reduced weight. On the other hand, new target data get higher weights for misclassified examples to intensify their impacts. In 2010, Yao et al. [41] proposed an extension of TrAdaBoost, called MultiSourceTrAdaBoost, by leveraging multiple sources of data for knowledge transfer. The author states that using a single source domain for knowledge transfer may lead to negative transfer and performance degradation due to the weak relationships between source and target. MultiSourceTrAdaBoost follows the same strategy as TrAdaBoost by applying weights to the source and target training data, except in the weak classifier selection. In each iteration of MultiSourceTrAdaBoost, a weak classifier is chosen based on the close relationships between source and target training data. Later, Liu et al. [16] designed a weighted resampling-based transfer learning framework (TrResampling) to improve the classification accuracy from TrAdaBoost. The algorithm resamples higher weights data in the source domain and adds this to the labeled target domain data. Then, the TrAdaBoost algorithm is applied for model building by adjusting source and target weights. Besides the resampling strategy, the study also assembled bagging-based [42] and MultiBoosting-based [43] transfer learning algorithms.

In addition to the boosting-based methods, various techniques exist to utilize the instances from source data. Tianyang et al. [44] proposed an instance-based deep transfer learning approach for image classification problems. The authors mainly pre-trained a model using source domain data and then applied it to labeled target training data. This strategy helps find the optimized target training set by estimating and removing the less influential target training data. Later, this optimized target data is used for building a new model or fine-tuning the previous pre-trained model. In 2016, Shuang et al. [45] proposed a source subset selection method by estimating the close relationships between source and target instances. The study employed an extension of Vovk’s conformity test for this purpose.

The Feature transfer learning setting assumes that there might be an inclusive relationship between source and target domains, and this approach tries to learn a new feature representation for the target domain. A cross-domain sentiment classification problem has been studied by Pan et al.  [46] through the feature alignment approach. The authors first identify the domain-independent and mutually dependent features and then build a spectral feature alignment (SFA) algorithm to reduce the difference between domain-specific features. In another work, Xia et al. [47] presented a feature ensemble method for sentiment classification where domain-independent features get higher weights, and domain-specific features get lower weights. The work of Oquab [48] employed a convolution neural network (CNN) architecture to transfer image representations trained on labeled large-scale source data to target tasks with a limited amount of data. As the image distributions are different for different domains, the study added a new adaptation layer to the CNN architecture of the target task. The key point in feature representation transfer learning is finding a good feature representation between domains with a different distribution. Pan et al. [49] proposed such a learning method named Transfer Component Analysis (TCA) for cross-domain WiFi localization and text classification.

Model transfer learning is also referred to as parameter-transfer learning. This approach finds out some shared parameters of the model for related source and target domains. Parameter-transfer methods are mainly effective for multi-task learning, where the adapted model is employed for the target tasks. TaskTrAdaBoost [41] is an extension of the TrAdaBoost algorithm for parameter-transfer based settings. The model identifies the shared parameters from different sources and target training part and reuses them to learn the target classifier. Another parameter-transfer method was proposed by Chattopadhyay [50] for detecting muscle-fatigue in various stages. The proposed framework relies on conditional probability distribution differences of multi-source data named Conditional Probability-based Multi-Source Domain Adaptation (CP-MDA). Differently, Segev et al. [51] proposed two model transfer learning algorithms: structure expansion/reduction (SER) and structure transfer (STRUT), based on a local transformation of a decision tree structure.

In our study, we focus on instance-based knowledge transfer. This approach is mainly motivated by importance sampling where relevant source domain data are re-weighted and/or target training subset selected before training the model. To the best of our knowledge, our study is the first of its kind to utilize such a knowledge transfer approach in crime prediction.

## Datasets

In this work, we consider crime incidents from three different cities (e.g., Halifax, Toronto, and Vancouver) for our cross-domain transfer learning approach. We consider Halifax as the target domain and Toronto and Vancouver cities as the source domains.

### Halifax data

We collected crime records from the Halifax Regional Police (HRP) department, which covers most of the Dissemination Areas (DAs) in Halifax Regional Municipality (HRM) in Nova Scotia. For the experiments, we explore crime incidents from January 2014 to December 2015. After deducting invalid and null records, we have a total of 18,818 and 17,744 records of crime incidents, respectively, for the years 2014 and 2015. We collected a total of 599 DAs for Halifax from the statistics Canada 2016 census. Besides raw crime data, each DA’s demographic data is extracted from the Canadian census analyser [52]. We also obtained Foursquare venue data, i.e., Point-of-interests (POI) and check-in information from April 2012 to January 2014 for Halifax city using Foursquare API [53]. The total collected POI venues for Halifax is 13,195. We have a total of 12,171 check-in data which indicate the user check-ins at different locations. Moreover, we retrieved streetlight information from the Street Light Vision (SLV) Central Management Server using SLV API. The data set contains 42,653 streetlight poles’ location information after filtering out the null values and invalid data.

### Toronto data

We used the Toronto Major Crime Indicators (MCI) 2014 to 2018 dataset as source data obtained from the public safety data portal of the Toronto police service  [54]. A total of 138,668 crime points are reported after excluding invalid and null points, where 2014 includes 26,507 records and 2015 consists of 26,796 records. On the other hand, there are 3702 DAs collected for Toronto from the statistics Canada 2016 census. Like the Halifax domain, we collected Foursquare POI and check-in data for Toronto using the same data source.

### Vancouver data

Besides the Toronto dataset, we explore crime occurrences for Vancouver as source data obtained from the Vancouver Open Data Catalogue. The data includes 24,573 crime records for the year 2014. According to the statistics Canada 2016 census, the city contains 993 Dissemination Areas. Like Halifax and Toronto, Vancouver’s demographic data was picked from the Canadian census analyser [52]. On the other hand, the Foursquare POIs and check-in data are missing for Vancouver.

The summary of the datasets for three different cities is given in Table 1. Tables 2 and 3 present the total number of venues for each POI category and the total number of check-ins for each time interval respectively, based on Halifax and Toronto cities. The number of check-ins is very low at time intervals 0 and 1, i.e., between 12 am and 6 am. The highest amount of check-ins occurs between 9 am and 9 pm. We removed the category ‘event’ from the experiment due to a small number of records and missing venue information.

**Table 1:** Details of the datasets used for the experiments

Dataset	Source	Total data		
		Halifax	Toronto	Vancouver
Historical crime data	Halifax Regional Police/Toronto public safety data portal/Vancouver Open Data Catalogue	18,818 (year-2014)	26,507 (year-2014)	24,573 (year-2014)
		17,744 (year-2015)		
Dissemination area data	Statistics Canada	599	3702	993
Demographic data	Canadian Census Analyser	599	3702	993
Streetlight data	Halifax Regional Municipality	42,653	–	–
Foursquare POI data	Foursquare	13,195	17004	–
Foursquare checkin data	Foursquare	12,171	123,397	–

**Table 2:** Total POIs for each category

POI category	Total POI count									
	Food	Residence	Arts and entertainment	College and university	Nightlife spot	Outdoors and recreation	Professional and other places	Shop and service	Travel and transport	Event
Halifax	1646	484	414	365	379	1112	2688	4525	1197	19
Toronto	4800	1197	664	513	851	1236	2813	3102	771	2

**Table 3:** Total check-ins for each time interval

	Total check-ins							
Time interval	0 (0-3)	1 (3-6)	2 (6-9)	3 (9-12)	4 (12-15)	5 (15-18)	6 (18-21)	7 (21-24)
Halifax	268	228	1575	2211	2482	2050	2479	878
Toronto	4474	1758	11604	16761	24744	22972	28451	12633

## Feature extraction

This section introduces the features extracted from multi-source urban data (Table 1) for future crime prediction. Based on the understanding of various aspects of urban crime factors, our extracted features are detailed in the following four subsections.

### Time-centric and historical features

**Fig. 2:**
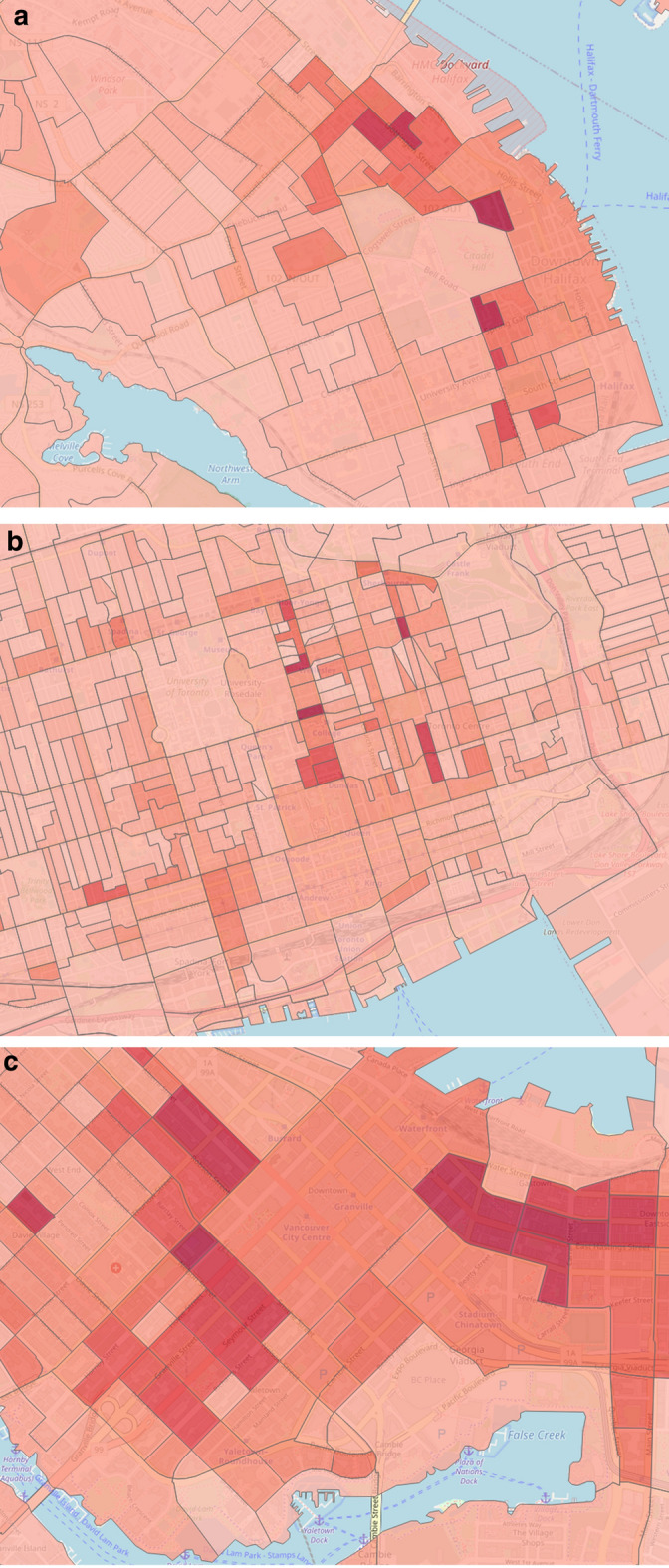
Crime densities by most observable DAs in **a** Halifax, **b** Toronto and **c** Vancouver

This feature category describes the static information of past crime incidents, which may have a relation with future crime events and the temporal crime information. Previous works found that both temporal and historical resources significantly influence future crime prediction [23, 25]. We examine a total of 12 features for this category. Temporal features include month, weekday, time interval, season, etc. By utilizing historical knowledge, we calculate crime frequency and density for each dissemination area (DA). We compute the crime density based on area, population, and season. Figure 2 pictures the crime densities of the downtown area from three different domains, such as Halifax (a), Toronto (b), and Vancouver (c).

### Location-based features: POI and streetlight

POI indicates specific venue information, including geographic location, which people find useful and may have a unique value due to its dynamism, such as a pub, a restaurant, or a train station. Besides specific POI type, we use 10 major POI categories (Table 2) identified by Foursquare. We mainly extract numeric features for each POI category, including the total number of venues and the densities of that category for each dissemination area. Regardless of the category, we also calculated total POIs in each DA.

**Fig. 3:**
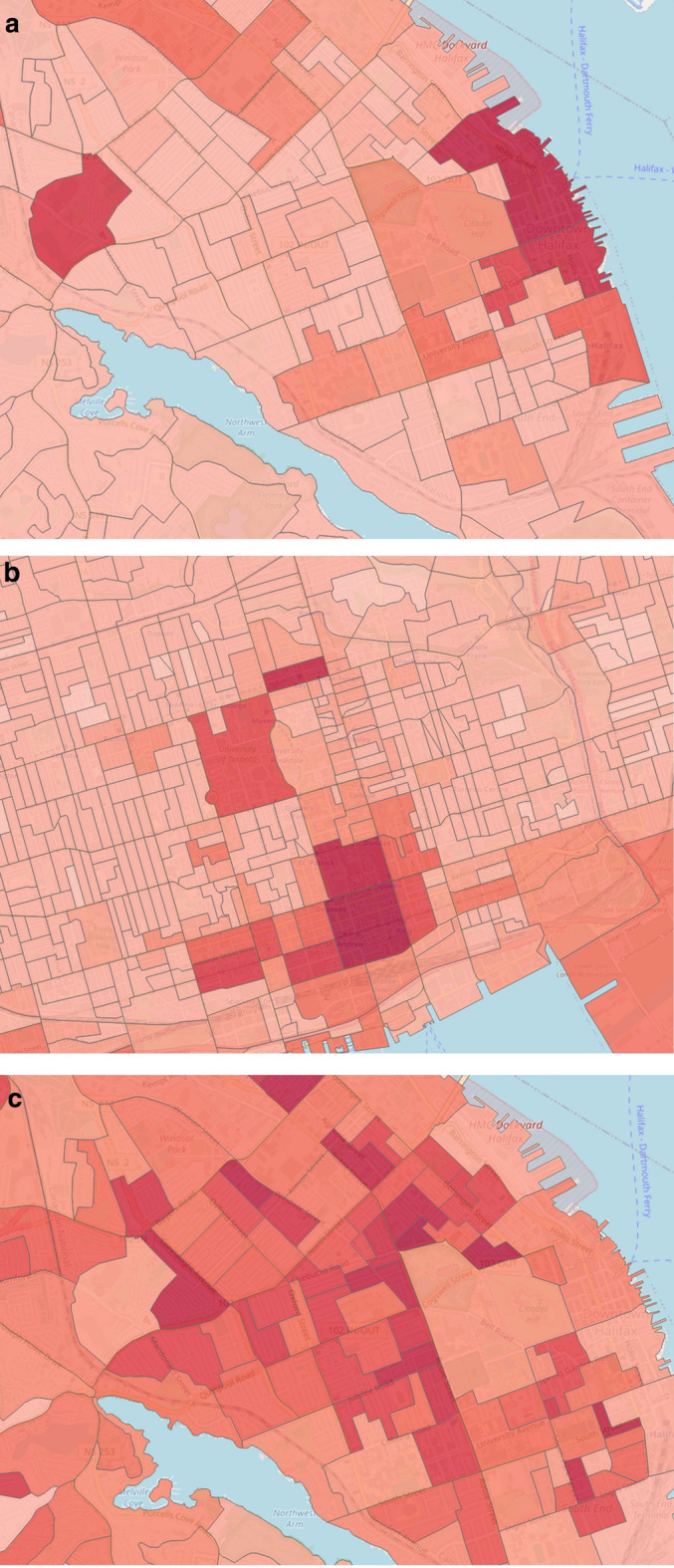
POI distributions and streetlight density by most observable DAs in Halifax and Toronto. **a** Halifax POI distribution, **b** Toronto POI distribution, **c** Halifax streetlight density

We extract 3 features from streetlight infrastructure data for crime prediction inspired by Bappee’s work [23]. Our identified features are (1) number of streetlight poles, (2) streetlight density, and (3) average distance from crime points to streetlight poles location. Figure 3a, b display the POI distributions for downtown Halifax and Toronto cities. On the other hand, Figure 3c shows the streetlight density for the downtown Halifax area.

### Demographic and socioeconomic features

**Fig. 4:**
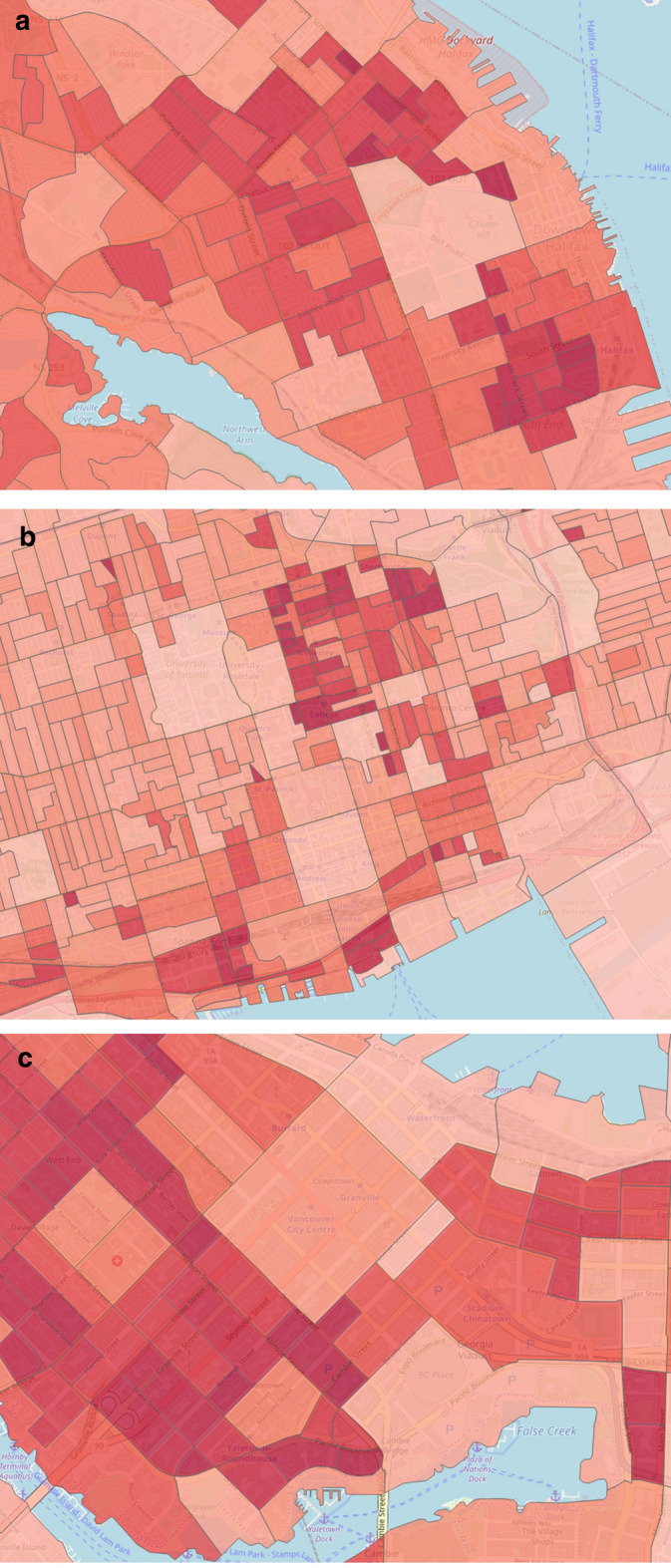
Population densities focusing on downtown areas for **a** Halifax, **b** Toronto and **c** Vancouver cities. The bin size for all three images is 100. The dark red color indicates high concentrated area and light red indicates the opposite

The 2016 census profile extracted from the Canadian census analyzer provides enormous demographic information for each dissemination block. Our analysis mainly includes the following features: total population, population density, income, residence information, mobility, commuting, age, sex, education, and race. The income profile measures the income statistics by different age groups, different household sizes, and total income groups for a specific reference period. Residence information measures the average household size, the number of persons in different households, total households by structure. The mobility feature measures the residential stability, i.e., if a resident is living in the same neighborhood for a long time or migrates from another place. The commuting profile provides information on how employed people of different age groups commute to the workplace and the time frame in which they leave for work. The race profile gives information about aboriginal identity and visible minorities for the population. In total, we extract 101 features per DA for this feature category. Figure 4 outlines the population density feature for three different domains.

### Dynamic features

In [23], the authors explored human mobility dynamic features using location-based social networks for a single domain, Halifax. For our study, we follow a similar feature extraction for cross-domain learning utilizing Foursquare data. This feature category mainly observes user check-in information when users reveal their location information and time with the Foursquare app. We construct 16 features for each dissemination area, including check-in density, visitor count, and region popularity. Check-in density for each DA and time slot is computed based on the total number of check-ins and that region’s area. Visitor count measures the location diversity by calculating the number of unique visitors for a particular region and time slot. Similarly, region popularity indicates a populous region or venue where many users check-in at a specific time interval. For instance, the region popularity feature for region r and time interval t is computed as follows:

1 $$\begin{aligned} \text {Region Popularity} (r,t) = \frac{\text {Total check-ins for region r at t}}{\text {Total check-ins for all regions at t}}, \end{aligned}$$

## Transfer learning on domain adaptation

### Multi-source domain adaptation

**Fig. 5:**
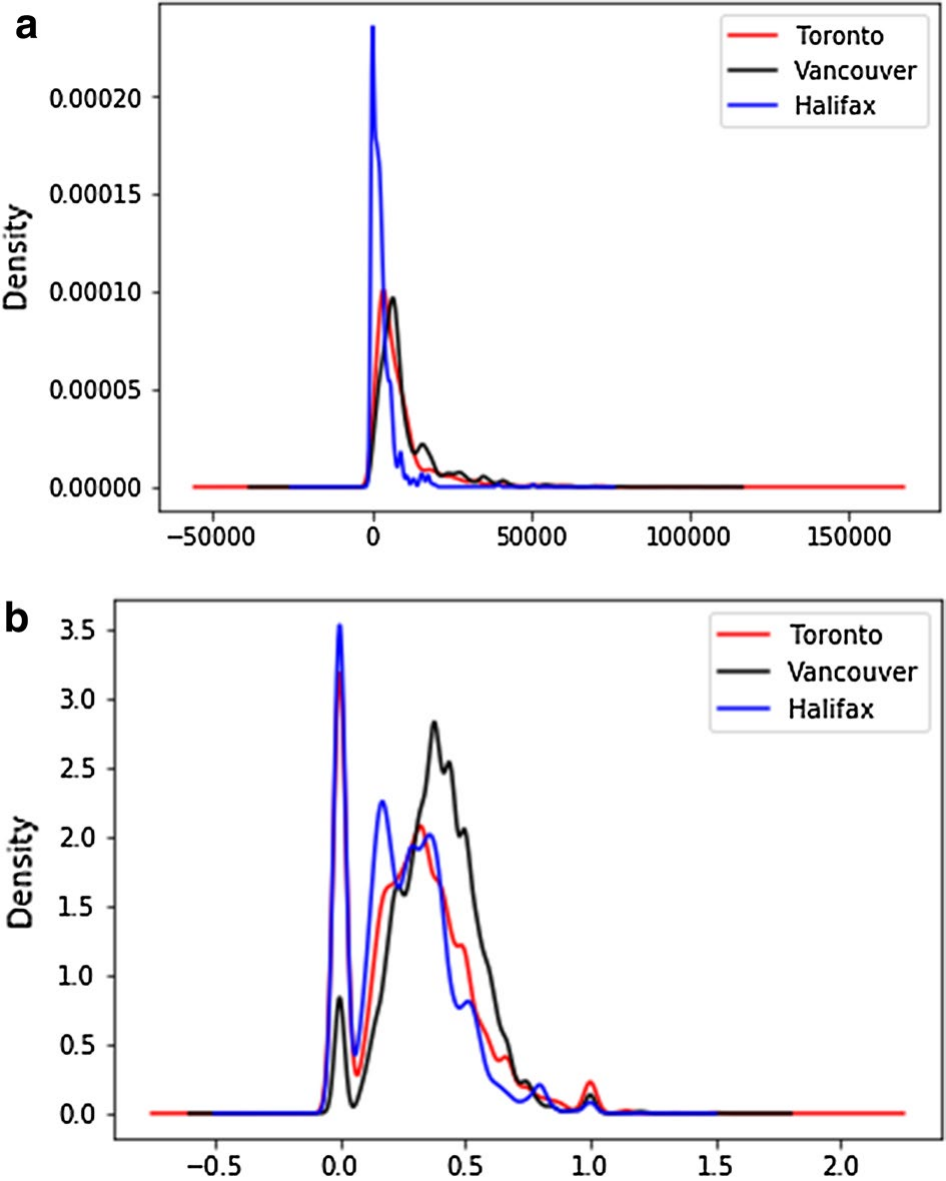
Distribution differences among three domains. **a** Population density. **b** Mobility migrants rate

In general, most of the existing transfer learning algorithms rely on a single source domain. However, transferring knowledge from only one source may lead to negative transfer occurrences and performance degradation. In transfer learning, negative knowledge transfer implies that the knowledge learned from the model by utilizing the source domain adversely affects the performance  [55]. The model confronts the negative knowledge transfer problem if the source and target domains are distantly related. The efficiency of transferring positive knowledge mainly depends on the relationship between source and target domains. Therefore, leveraging multi-source domains helps find the related source and target domains and reduces the possibility of negative transfer by importing knowledge from the closely related source. Here, the purpose of the multi-source domain adaptation is to align the labeled data from multiple sources, e.g., Toronto and Vancouver, where the distributions are also different, as well as enabling the data to be processed together along with the labeled target data (Halifax). Before approaching knowledge transfer among domains, we analyze three domains’ distribution differences in feature space and label space. Afterward, the domains are adapted to an individual representation by reducing the distances among them. The distributions of three different cities based on population density and mobility migrants rate are shown in Fig. 5a, b, respectively. The distributions of population density are nearly related among the three domains. However, for the mobility migrants rate, the distributions between Halifax and Vancouver domains are roughly different.

To illustrate the distribution difference between source and target domains for individual features we use the Kullback–Leibler divergence (KL-Divergence) method [56]. Table 4 provides the distribution differences between the Halifax domain and the two source domains (Toronto and Vancouver) for different Demographic features. The larger value indicates that the distribution of the source and target domains for the specific feature is very different. For instance, the distribution of Toronto and Vancouver highly differs from Halifax for the features ‘total visible minority population’ and ‘Journey to work by public transit’.

**Table 4:** KL-divergence between Halifax and the two source domains

Features	Distribution Difference-Halifax						
	Population density	Mobility migrants’ rate	Low-income rate	Private households 1 person	Journey to work between 8 am and 9 am	Total visible minority population	Journey to work (public transit)
Toronto	0.146	0.031	0.012	0.031	0.116	1.421	1.567
Vancouver	0.382	0.330	0.120	0.126	0.4	2.165	1.136

For domain adaptation, we propose to apply two approaches: (i) local min–max normalization and (ii) global min–max normalization, motivated by the work of cross-building energy forecasting [57]. The general min-max normalization formula is represented as:

2 $$\begin{aligned} N(x_{ij}) = \frac{x_{ij}-min(X_{j})}{max(X_{j}) - min(X_{j})}, \end{aligned}$$

where, $i = 1,\dots , n$; $j = 1,\dots , m$; *n* is the total number of instances; and *m* is the total number of features.

Local min-max normalization focuses on the relative relationship between an input feature ($X_j$) and an output variable (*Y*). This approach considers each domain locally and uses the local minimum and maximum values for normalization. Suppose, *N*() is a normalization function and we have 2 source domains ($S = 2$). For local normalization, we calculate $N(X_{j}^1)$ and $N(X_{j}^2)$ separately.

Global min-max normalization gives particular attention to the absolute relationship between $X_j$ and *Y*. This approach considers each domain as a subset of a global domain (D) where the feature ($X_j$) of each domain belongs to a superset *J*. We calculate global normalization as $N(X_{j}^D,J)$.

### Transfer learning settings

**Fig. 6:**
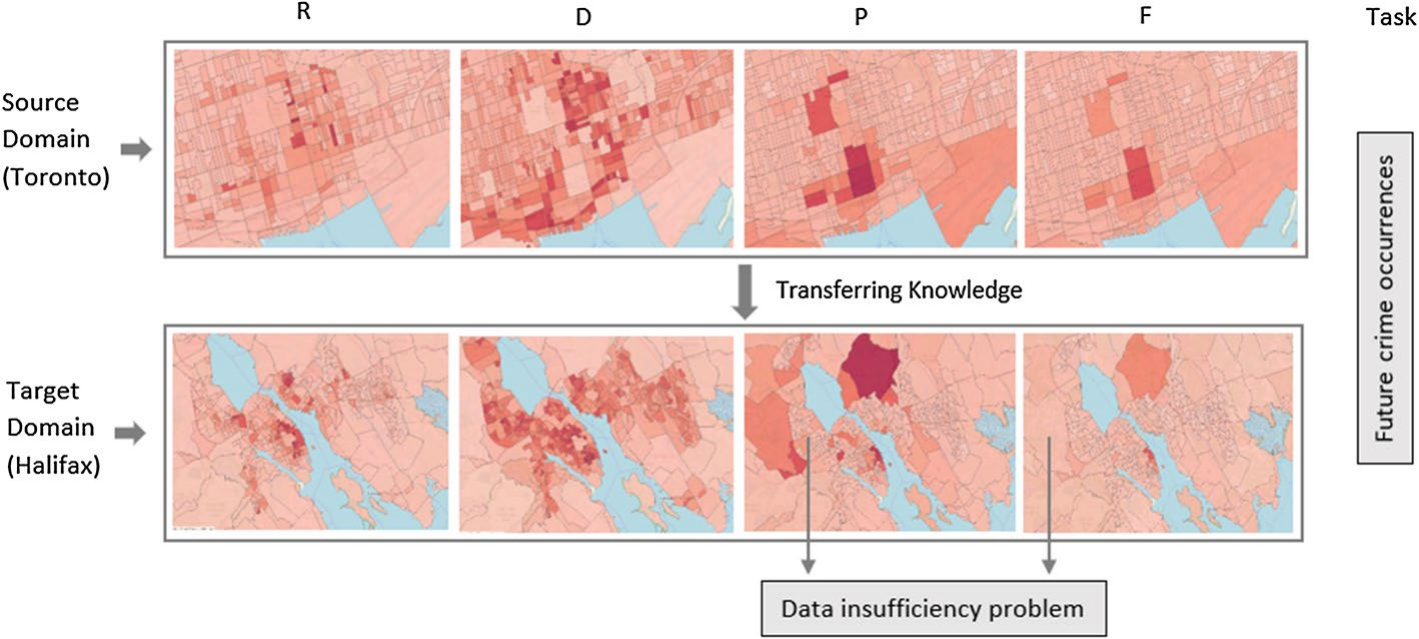
Transferring knowledge from Toronto (source) to Halifax (target). R: Raw features, D: Demographic features, P: Foursquare POI features and F: Foursquare dynamic features

For knowledge transfer, instead of using single modality data, we utilized all feature categories extracted in the “Feature extraction” section with multimodal characteristics. Figure 6 exhibits an example of transferring knowledge from Toronto to Halifax city with four feature categories for the task of crime occurrence prediction. Here, R, D, P, and F indicate raw, demographics, Foursquare POI, and Foursquare dynamic feature categories, respectively. In this figure, we assume that the data from Foursquare POI (P) and dynamic (F) sources are not sufficient for Halifax city compared to Toronto. In such situations, we can learn from the source domain, Toronto, regarding the inherent relationships between people’s movement in any specific POI and crime occurrences. Later, we can use this knowledge to predict Halifax’s crime occurrences based on the dynamic features even though there is a data insufficiency problem.

We built six different models of knowledge transfer based on cross-domain data fusion. Model 1 is implemented based on the available target training data. However, there might be an overfitting problem if the available target training set is limited. Given no labeled data from the target domain, model 2 is built upon the Toronto source only. Similarly, model 3 is based on the Vancouver data only. On the other hand, model 4 belongs to the Toronto source data and available Halifax data. Model 5 includes Vancouver source data along with the available data from the Halifax target. Model 6 imports knowledge from both the Toronto and Vancouver sources to find the relatedness with the target domain.

The model representation for multi-source data is given below:

$$\begin{aligned}&\text {Model} \ 1 : H \rightarrow H \\&\text {Model} \ 2 : T \rightarrow H \\&\text {Model} \ 3 : V \rightarrow H \\&\text {Model} \ 4 : T \cup H \rightarrow H \\&\text {Model} \ 5 : V \cup H \rightarrow H \\&\text {Model} \ 6 : T \cup V \cup H \rightarrow H. \end{aligned}$$

Here, H, T, and V indicate Halifax, Toronto, and Vancouver domains, respectively. We tested models 2 and 4 using all feature categories extracted before except streetlight features due to the lack of this category in the Toronto domain. Models 3, 5, and 6 are implemented based on raw (R) and demographic (D) features only. For our current study, foursquare feature categories (P and F) are unavailable for the Vancouver domain. The feature representation for different models is given below:

$$\begin{aligned}&\text {Model} \ 1 : R \cup D \cup S \cup F \cup P \\&\text {Model} \ 2 : R \cup D \cup F \cup P \\&\text {Model} \ 3 : R \cup D \\&\text {Model} \ 4 : R \cup D \cup F \cup P \\&\text {Model} \ 5 : R \cup D \\&\text {Model} \ 6 : R \cup D. \end{aligned}$$

### Seasonal-subset selection

**Fig. 7:**
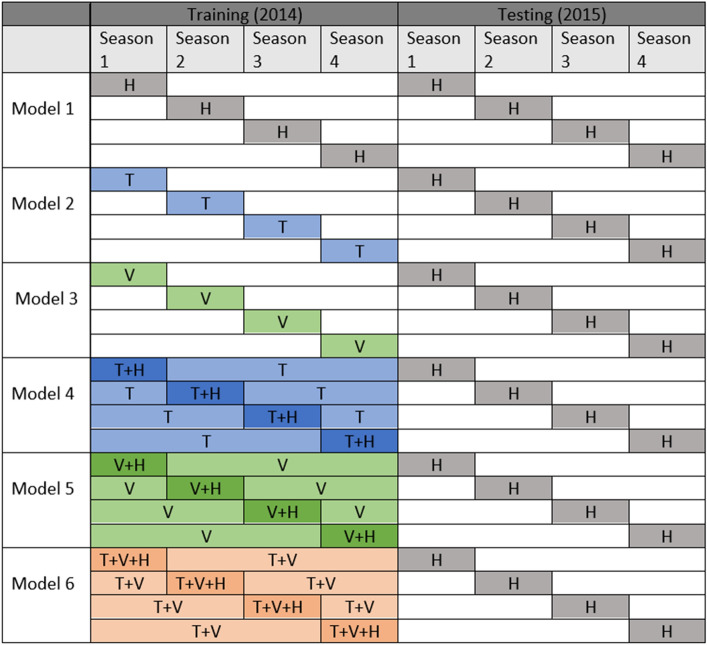
Six different scenarios based on seasonal perspective

Besides source domain data, our model representation relies on a small amount of target domain data that we call the target training set. Our first approach is selecting the target training set that focuses on seasonal aspects since we want to consider the impact of seasonality on crime occurrences and the seasonal target predictive set. Figure 7 shows six different scenarios based on seasonal perspectives. Model 1, which only considers a small amount of target (Halifax) data for training, uses consecutive seasons for knowledge transfer. Similarly, models 2 and 3 utilize consecutive seasons from Toronto and Vancouver domains, respectively, for model building. However, it assumes there is no available target training data for the study. On the other hand, model 4 transfers consecutive seasonal instances from the Halifax domain and all 4 seasons from the Toronto domain. Likewise, in model 5, all successive seasonal instances from the Halifax domain and all 2014 data from the Vancouver domain are used for instance transfer. In model 6, we assume all seasonal instances from Toronto and Vancouver domains are available along with the consecutive Halifax seasonal instances.

## Experimental setup and result analysis

### Data preparation

In this section we describe the data indexing, mapping and integration. We group and index crime occurrences based on the DA where they happened, the year, month, day of the week, and the time interval of the day. We partition a day into 8 three-h time intervals. The final size of the data index for each year based on Halifax city is 402,528 (12*7*8*599).

After data indexing, we map all crime records to one of the 599 DAs collected for Halifax from the statistics Canada 2016 census, based on their geographic location. However, most of the data (around 87%) are labeled with ‘no crime’ in the data index as crime events are not frequent. We labeled an observation as ‘no crime’ when there was no crime information for a specific time slot. To address the data imbalanced issue, we apply a well-known machine learning imbalanced learn strategy on ‘no crime’ records to obtain a more balanced dataset [58]. We choose the random under-sampling technique to randomly select a subset of instances from the majority class (no crime) of the training data. We achieve equal class distribution by applying this approach. On the other hand, removing informative data points from the majority class may direct to a biased dataset and cause performance degradation. However, we are employing the random under-sampling technique on artificially generated ‘no crime’ records only. Furthermore, we have sufficient data points from the minority class to fit the model. This approach is relevant for our study even with the imbalanced class distribution.

Similar to crime data, we map each demographic profile, streetlight pole, POI venue, and check-in location to the corresponding dissemination area using their geographic coordinates. Later, we join all groups of data with crime data by following our formatted index. We use GeoPandas library (https://geopandas.org/) and QGIS (Quantum GIS) tool (https://qgis.org/en/site/) to conduct these spatial operations.

We follow the same data preparation techniques for Toronto and Vancouver domains. We map all criminal records and other collected data records from Toronto and Vancouver domains to one of the 3702 and 993 DAs, respectively, based on their geographic location.

As discussed in “Datasets” section, we use Halifax data as target domain data where a subset of the year 2014 data is used for training and 2015 data are for testing. On the other hand, 2014 Toronto and Vancouver data are employed for training as source data. After mapping and under-sampling the no criminal records for training data based on each time interval and DA, we have a total of 53,014 and 49,146 records for Toronto and Vancouver domains, respectively. The sizes of training and testing data are summarized in Table 5.

**Table 5:** Training and testing splits after data sampling and mapping

Domain	Training data		Testing data	
	Crime	No crime	Crime	No crime
Halifax	18,818	18,818	17,744	384,112
Toronto	26,507	26,507	–	–
Vancouver	24,573	24,573	–	–

### Prediction model

Motivated by the fact that ensemble learning methods can adopt generalization [59] on different domains where the distributions are also different, we consider using ensemble-based machine learning methods for our transfer learning phase. We mainly focus on the Gradient Boosting (GB) [60] classifier to run the experiment under the instance-transfer learning paradigm. The key reasons to choose GB over other popular machine learning algorithms are its enticing qualities and the challenging crime data characteristics. Other than the generalization capability on unseen data, GB can handle nonlinear relationships among diverse sources of data. Moreover, the model does not require large datasets to evade overfitting problems and maximum effort and attention for data cleaning and preparation. We applied a randomized grid-search technique to find out the optimized parameter settings for the selected classifiers. We compare our results with a popular bagging ensemble method: Random Forest (RF), and well-known boosting ensemble-based transfer learning methods: TrAdaBoost and TrResampling.

## Results and discussion

For the methods’ performance evaluation, we analyze the AUC (Area Under the ROC Curve) and Gmean scores. Table 6 evaluates the performance metrics of GB classifier and RF classifier for six different models (described in section “Transfer learning settings”). This evaluation is based on the season-specific training set.

**Table 6:** Performance evaluation based on six different models

Season	Model	Random forest		Gradient boosting	
		AUC (%)	Gmean (%)	AUC (%)	Gmean (%)
1	1	69.69	69.25	69.25	68.95
	2	64.34	63.39	65.17	65.14
	3	63.18	62.61	63.17	62.69
	4	69.55	69.13	70.25	69.73
	5	70.00	69.48	70.05	69.74
	6	69.69	69.11	70.10	69.71
2	1	69.61	69.57	69.25	69.24
	2	64.47	64.35	65.04	64.94
	3	63.09	62.10	63.74	63.54
	4	69.54	69.50	70.63	70.59
	5	69.41	69.35	70.35	70.35
	6	69.28	69.20	70.52	70.52
3	1	67.57	67.55	67.54	67.49
	2	63.74	63.65	63.86	63.66
	3	62.89	62.72	62.54	62.06
	4	67.42	67.41	68.70	68.68
	5	67.57	67.56	67.99	67.87
	6	67.52	67.51	68.12	68.04
4	1	68.76	68.68	68.12	68.08
	2	64.20	64.08	64.80	64.54
	3	62.85	62.84	62.41	62.37
	4	68.65	68.54	69.23	69.09
	5	68.79	68.66	68.99	68.96
	6	68.80	68.66	69.11	69.07

Though RF and GB classifiers exhibit similar patterns, GB performs better than RF for most of the models. According to the GB classifier results, model 1 reports 69.25% AUC and 68.95% Gmean scores based on season 1. The rest of the seasons also show a similar performance except for season 3. On the other hand, model 2, trained with Toronto source data only, results in low AUC and Gmean scores for almost all seasons compared with model 1. For instance, model 2 degrades approximately 4% AUC (65.17%) and Gmean (65.14%) scores for season 1. Likewise, model 3, built on Vancouver data source only, reduces approximately 6% performance for AUC and Gmean scores. The performance loss may happen due to the weak connections between Vancouver and Halifax domains. Moreover, the outcomes highlight the significance of comprising accessible target-specific instances for model building. Model 4, which incorporates the target training set with Toronto source data, exhibits the best performance among all six models. For instance, model 4 presents around 1%, 5%, and 7% performance improvement with AUC (70.25%) and Gmean (69.73%) scores compared to models 1, 2, and 3, respectively for season 1. It tells that adding instances from Toronto data to Halifax data promotes positive knowledge transfer. Model 5 and 6 show almost similar results as model 4 for each season, i.e., adding Toronto and Vancouver sources together does not help to enhance performance. The reason might be (1) the data scarcity of foursquare feature categories in the source domain and/or (2) negative knowledge transfer due to the distant relationships between source and target domains.

Table 7 compares the AUC and Gmean scores achieved from GB classifier with two base transfer learning algorithms: TrAdaBoost and TrResampling for model 4. The GB classifier’s AUC scores show approximately 4.8% and 3.5% improvement compared to TrAdaBoost and TrResampling methods, respectively, for season 1. Similarly, for Gmean our proposed algorithm promotes 4.3% against TrAdaBoost and 4.8% against TrResampling. The rest of the seasons also show similar patterns. The most probable reasons for base learners to degrade performance are that TrAdaBoost is sensitive to the quality of instances from the different distributions and cannot handle multi-source different data distributions. Like TrAdaBoost, TrResampling faces the same kinds of issues, i.e., the negative knowledge transfer problem, though it performs slightly better than TrAdaBoost.

**Table 7:** AUC and Gmean scores based on Toronto and Halifax data (model 4)

Model 4	Gradient boosting		TrAdaBoost		TrResampling	
Season	AUC (%)	Gmean (%)	AUC (%)	Gmean (%)	AUC (%)	Gmean (%)
1	70.25	69.73	65.46	65.40	66.70	64.88
2	70.63	70.59	65.46	65.16	66.50	65.14
3	68.70	68.68	64.17	63.80	64.20	63.40
4	69.23	69.09	65.02	64.94	65.30	64.58

The results of model 5 learned from Vancouver and Halifax sources are shown in Table 8. For TrAdaBoost, adding available target instances (season 1) with Vancouver source data reveals 4.9% and 4.6% performance drop respectively with AUC and Gmean metrics compared to GB classifier. On the other hand, TrResampling drops approximately 3.5% and 4.8% performance than GB based on AUC and Gmean scores. Table 9 evaluates the results from multi-source (Toronto and Vancouver) data based on model 6. Model 6 reaches similar patterns for TrAdaBoost and TrResampling. In summary, Gradient Boosting performs better than base algorithms for both models 5 and 6. The reasons for this performance degradation are the same as discussed for model 4 i.e., models’ sensitivity on data and lack of handling multi-source data.

**Table 8:** AUC and Gmean scores based on Vancouver and Halifax data (model 5)

Model 5	Gradient boosting		TrAdaBoost		TrResampling	
Season	AUC (%)	Gmean (%)	AUC (%)	Gmean (%)	AUC (%)	Gmean (%)
1	70.05	69.74	65.17	65.15	66.6	65.17
2	70.35	70.35	65.59	65.49	66.2	65.11
3	67.99	67.87	63.70	63.57	64.7	63.56
4	68.99	68.96	64.37	64.26	65.0	63.82

**Fig. 8:**
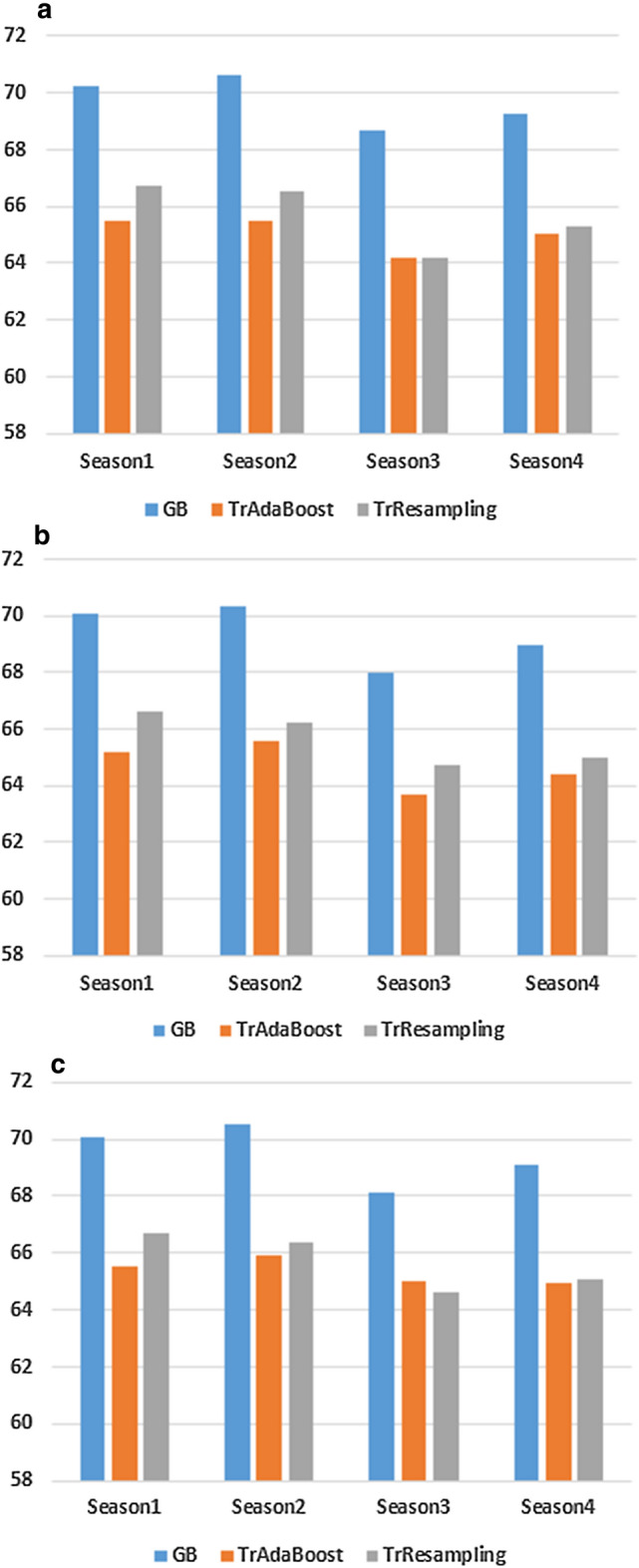
Comparison of AUC scores. **a** Model 4 seasonal subset. **b** Model 5 seasonal subset. **c** Model 6 seasonal subset

Figure 8 depicts the overall picture of models 4, 5, and 6 applying seasonal subset selection based on AUC scores. We observe almost similar patterns for three different models i.e., the performance after accumulating Toronto and Vancouver sources together (model 6) does not improve over models 4 and 5. It is because we encounter a data scarcity problem for some feature categories in the source domain. Also, combining multi-source domain with distant relation cannot promote positive knowledge.

**Table 9:** AUC score and Gmean based on multisource data (model 6)

Model 6	Gradient boosting		TrAdaBoost		TrResampling	
Season	AUC (%)	Gmean (%)	AUC (%)	Gmean (%)	AUC (%)	Gmean (%)
1	70.10	69.71	65.53	65.52	66.70	64.98
2	70.52	70.52	65.98	65.84	66.40	64.90
3	68.12	68.04	65.00	64.88	64.60	63.40
4	69.11	69.07	64.95	64.92	65.10	63.65

### Discussion based on coefficient study

As we have a categorical target variable (crime and no crime), we use logistic regression to understand the correlation between continuous input variables and the categorical target variable. We measure the feature importance of multi-source domain through the coefficient values taken from the logistic regression model. Table 10 shows the top 19 features with positive and negative coefficients. Features with positive and negative coefficients indicate positive and negative correlations with crime respectively. From the table, we find that different POI features such as shop service count, venue count, food count, and travel transport count are highly correlated with crime. However, we have a data scarcity problem for these features in the Vancouver domain. Similarly, another important feature, streetlight density is also absent in the Toronto and Vancouver domains. This is one of the reasons for Toronto and Vancouver sources not exhibiting significant performance improvement especially when we compare models 2 and 3 with model 1 (Table 6). Besides, if we compare the feature distribution (Table 4) and feature coefficient, the KL-divergence value for some correlated features e.g., ‘total visible minority population’ and ‘Journey to work by public transit’ are high. This is also a bottleneck for other models to surpass model 1.

**Table 10:** Top 19 features with positive and negative coefficients

Feature	Coefficients	Feature	Coefficients
Population	3.192	Outdoor and recreation count	1.380
Shop Service count	2.940	Total visible minority population	1.340
Private households (1 person) rate	2.284	Mobility movers rate	1.271
Venue count	2.099	Journey to work (public transit)	1.248
Visitor count	1.822	Nightlife count	$-1.265$
Food count	1.766	No. of person in private households (rate)	$-1.319$
Travel transport count	1.509	Arts & entertainment count	$-1.471$
Journey to work (between 12pm & 5am)	1.472	Journey to work (between 8 am & 9 am)	$-1.561$
Streetlight density	1.470	Population density	$-1.843$
Professional and other places density	1.405	–	–

## Conclusions and future work

We implemented a data-driven approach for crime prediction by investigating several feature combinations for cross-domain learning in this work. As it is challenging to prepare enough labeled training data based on a small city like Halifax, we examine multi-source domain adaptation by leveraging knowledge from two other domains: Toronto and Vancouver. We applied instance-based transfer learning techniques for transferring knowledge between source and target domains. For instance, we proposed different settings based on season-specific subset selection with cross-domain data fusion. We mainly focused on ensemble learning methods for cross-domain learning because of its generalization ability with new data. We evaluated the GB and RF classifiers for all proposed setups and compared the results with two base transfer learning algorithms: TrAdaBoost and TrResampling. Based on our experiments, the ensemble-based GB classifier improves the AUC scores by average 4% with TrAdaBoost and 3.8% with TrResampling for multi-source data. From all the experiments on instance transfer learning, we can conclude that the GB classifier works better when available target-specific instances are added to the Toronto source.

We have several future works and ideas regarding multi-source domain adaptation in crime pattern detection and prediction. For example, identifying specific types of crime that might happen in the near future is our immediate concern for cross-domain learning. Our analysis based on the Halifax domain shows that different types of crime exhibit different spatial and temporal distributions. Correspondingly, behavioral patterns, mobility, and networking might be different for individual crime types. Therefore, investigating the prediction performance and the significance of individual features for cross-domain study capturing various crime categories is of great importance. In addition, we intend to appraise our research of cross-domain learning for crime prediction at a wider level in the future, such as the country level, instead of only focusing on the city level. In our study, we mainly focus on the instance transfer learning problem. For feature representation, we highlight the common features among source and target domains. However, learning a good feature representation and transferring that knowledge to the target domain is simultaneously essential. Particularly, when a full structure is missing for any specific modalities (e.g., POI data is missing in the Vancouver domain), rather than picking just common features learning feature knowledge from the other domains will be advantageous. Adding particular types of crime might require different but related tasks for source and target domains. In such cases, exploring parameter-transfer and a relational-knowledge transfer would be interesting. Investigating discrimination in socially-sensitive decision records is state-of-the-art research to avoid biased classification learning. In a societal context, discrimination indicates unjust or unequal actions of people based on preconceptions. If protected attributes such as gender or race have an explicit contribution to decision-making or dependency on other correlated features, discrimination may also occur in the trained model. As we are using real-world crime data for our study, investigating and preventing discrimination is highly crucial before decision-making. We plan to investigate a pre-processed discrimination prevention technique on our crime data by following the idea from Calmon et al. [61]. The study includes three properties: discrimination control, distortion control, and utility preservation. Apart from the pre-processed discrimination prevention, we will also explore the post-processing approach [62] for discrimination prevention.

## Data Availability

Demographic data is available at: http://datacentre.chass.utoronto.ca/cgi-bin/census/2016/displayCensus.cgi?year=2016&geo=da. Dissemination area data is available at: https://www12.statcan.gc.ca/census-recensement/2011/geo/bound-limit/bound-limit-2016-eng.cfm. Toronto crime data is available at: https://data.torontopolice.on.ca/search. Vancouver crime data is available at: https://www.kaggle.com/wosaku/crime-in-vancouver and https://opendata.vancouver.ca/pages/home/. Halifax crime data and streetlight data are restricted and not available publicly. The Foursquare data is available at: https://sites.google.com/site/yangdingqi/home/foursquare-dataset.
